# Challenges in diagnosis and management of invasive ductal carcinoma in axillary ectopic breast tissue: a case study

**DOI:** 10.1093/jscr/rjae531

**Published:** 2024-08-26

**Authors:** Alsadig Suliman, MagdAlden Osman, Siddig Ali, Sara Hussein, Reem Mohamed Osman, Enas Tageldin, Lobna E Ali

**Affiliations:** Department of General Surgery, Sudan Medical Specialization Board, Isbitalia Street, Downtown, Khartoum, Khartoum, Sudan; Department of General Surgery, Alwaledeen Specialized Hospital, Hospital Street, Souq District, Wad AL Naeem 21114, Sudan; Department of General Surgery, Sudan Medical Specialization Board, Isbitalia Street, Downtown, Khartoum, Khartoum, Sudan; Department of General Surgery, Sudan Medical Specialization Board, Isbitalia Street, Downtown, Khartoum, Khartoum, Sudan; Department of General Surgery, Alneelain University, Zubeir Pasha Street, Downtown, Khartoum 1115, Sudan; Department of General Surgery, Sudan International University, Alsiteen Street, Azhari District, Khartoum 1115, Sudan; Department of Histopathology, Port Sudan Ahlia College, Transit District, Transit Street, Port Sudan, Sudan

**Keywords:** ectopic breast tissue, invasive ductal carcinoma, axillary breast cancer, wide local excision

## Abstract

Ectopic breast tissue (EBT) is breast tissue located outside the normal anatomic boundaries of the breasts, developing due to incomplete embryological regression of the mammary ridges. EBT can develop anywhere along the milk line, with the axilla being the most common site. While generally benign, EBT can undergo malignant transformation. This case report discusses a 24-year-old female with locally advanced invasive ductal carcinoma in the axillary EBT, highlighting its clinical presentation, diagnostic process, and management in a resource-limited setting. The patient underwent wide local excision and axillary lymph node dissection followed by adjuvant chemotherapy and radiotherapy, achieving a favorable short-term outcome. This case underscores the importance of considering EBT in differential diagnosis of axillary masses and the need for tailored treatment strategies in such settings.

## Introduction

Ectopic breast tissue (EBT), also known as accessory breast tissue, refers to the presence of breast tissue located outside the normal anatomical boundaries of the breasts [1]. EBT develops due to incomplete embryological regression of the mammary ridges, which extend from the axilla to the vulva [2, 3]. It is present in 2%–6% of the population and, like normal breast tissue, has the potential to develop malignancy [4, 5]. Although malignancies in EBT are rare, they tend to have a progressive clinical course. The incidence of EBT carcinoma is 0.3%–0.6% of all breast cancers, with the axilla being the most common site [6]. We present a unique case of invasive ductal carcinoma (IDC) in the EBT of the axilla, highlighting the diagnostic and therapeutic challenges associated with this condition.

## Case report

**Figure 1:**
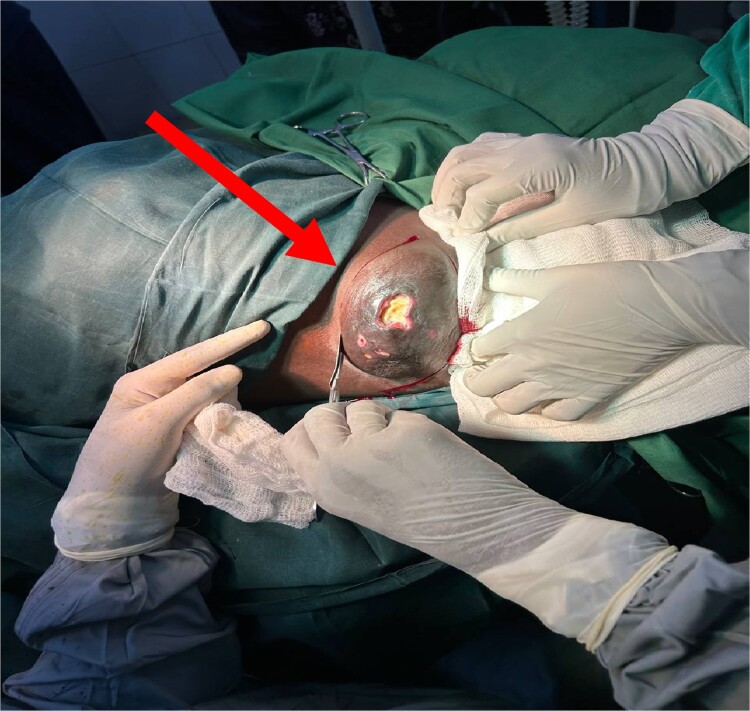
Beginning of surgery (WLE) at left axilla with ulcerated prominent ectopic breast tissue (indicated by an arrow).

A 24-year-old female, unmarried and a nonsmoker, presented with a painless mass in her left axilla that had been developing for approximately six months. She also reported significant weight loss and fatigue but had no family history of breast cancer. On physical examination, both breasts and the right axilla were normal. However, the left axilla revealed a hard, mobile lump measuring 12.6 × 8.4 × 4.5 cm with hyperpigmented and ulcerated skin. In addition, palpable, painless left axillary lymph nodes (ALNs) were observed (Fig. 1).

**Figure 2:**
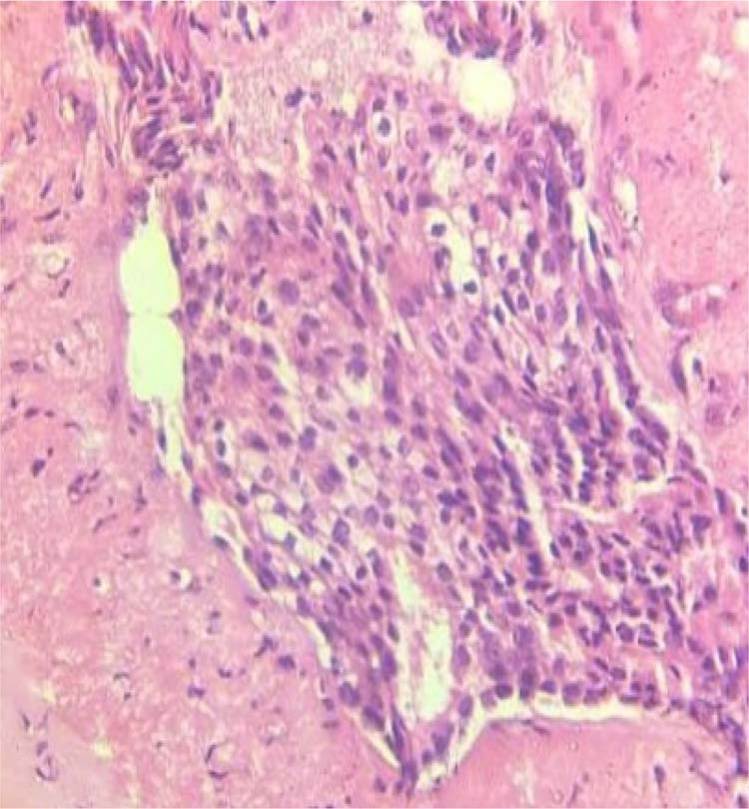
H&E stain slide shows histopathological features of IDC.

An ultrasound of the left axilla revealed a mass measuring 12.6 × 8.4 × 4.5 cm, located near the anterior border of the latissimus dorsi muscle and inferior to the axillary vein. The mass exhibited heterogeneous echotexture and irregular margins, suggestive of malignancy. Additionally, three enlarged ALNs with irregular borders were identified. A core needle biopsy of the mass, guided by ultrasound, confirmed the presence of IDC (Fig. 2). Furthermore, an ultrasound-guided biopsy of the suspicious ALNs confirmed malignancy.

Immunohistochemical analysis of the axillary mass biopsy revealed estrogen receptor and progesterone receptor positivity, while human epidermal growth factor receptor 2 was negative. Thoracoabdominal CT showed no secondary localizations, and whole-body bone scintigraphy detected no bone metastases. The overall stage was determined to be T4bN1M0 (Stage IIIB).

**Figure 3:**
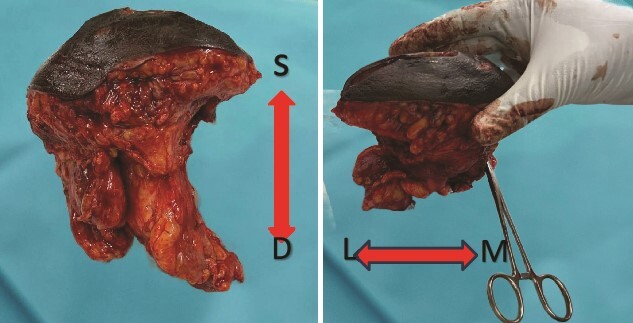
Gross finding appearance of the EBT specimen showed a solid mass with clear margins. **S**: superficial margin; **D**: deep margin; **M**: medial margin; **L**: lateral margin.

A multidisciplinary team determined the patient to be a candidate for neoadjuvant chemotherapy (NAC) followed by surgical management and adjuvant chemotherapy (AC). However, due to the unavailability of chemotherapy at the time of diagnosis, surgical management proceeded after careful patient counseling. A wide local excision (WLE) of the mass with levels I and II axillary lymph node clearance (ALNC) was performed (Fig. 3). Histopathology reported a Nottingham grade III IDC with infiltrative borders and dense marginal lymphatic infiltration. The tumor was totally excised with 10 out of 15 soft white lymph nodes showing extensive metastatic deposits. The patient did not suffer any complications postoperatively. Upon follow-up after three weeks, the patient was in good health. Postoperatively, she received four of the six planned cycles of adjuvant chemotherapy; the fourth cycle was canceled due to neutropenia. She also received hormonal therapy and radiotherapy as per the treatment plan.

## Discussion

EBT results from the failure of complete regression of the mammary ridges during embryological development. Axilla is the most common location for EBT, accounting for 58% of reported cases [6]. Other locations include the parasternal line, sub clavicular area, sub-mammary region, and vulvar region [7]. EBT may contain only glandular tissue or be associated with a nipple-areola complex [8]. Hormonal regulation influences its development, and it may become apparent during puberty and pregnancy, similar to normal breast tissue [9]. EBT is subject to the same pathologies, including pain, inflammation, fibroadenoma, and cancers [6]. However, the exact incidence of primary EBT carcinoma and the rate of malignant transformation remain unknown [2]. Accurate diagnosis of axillary EBT carcinoma is crucial as it provides precise staging information for patients with concurrent ipsilateral breast cancer [10]. IDC is the most frequently reported pathological type of EBT cancer, although other types such as medullary, lobular, and phyllodes have also been reported [6].

Managing EBT cancer is consistent with the guidelines for pectoral breast cancer, involving a multidisciplinary approach and standard triple therapy: surgery, systemic therapy, and radiation, depending on the staging and tumor biology [7]. Imaging studies like breast MRI, US, and mammography are vital for assessing EBT cancer [6]. Fine-needle aspiration cytology can aid in diagnosing malignancy, but distinguishing between EBT cancer and lymph node metastasis from occult primary lesions can be challenging. Histologically, the presence of adjacent normal breast tissue (ducts and lobules) and lack of lymphoid tissue confirm the diagnosis of EBT cancer and exclude metastatic lymph nodes [11]. According to lymph node guidelines, using ultrasound-guided biopsy of suspicious ALNs is essential in confirming malignancy before undertaking ALNC [12].

This case involves a young patient with locally advanced IDC of axillary EBT. Despite resource limitations, WLE with ALNC followed by AC and radiotherapy contributed to a favorable short-term outcome. The choice of treatment modalities was guided by the aggressive nature of the tumor, ALNs involvement, and the need to achieve optimal disease control.

In modern breast surgery, NAC would have been the gold standard prior to surgery for a premenopausal patient with metastatic ALNs. NAC is significant in reducing tumor volume to increase the breast-conserving rate. It is crucial for downgrading locally advanced inoperable patients to provide surgical opportunities [13]. On the other hand, AC was initiated to target potential micrometastases and systemic spread, aligning with recommendations for cases with adverse prognostic features. The patient's response highlights the critical role of surgical intervention in such cases [14]. Adjuvant radiotherapy was administered to enhance local control and reduce the risk of recurrence, in accordance with recommendations despite existing controversies regarding its use in ipsilateral disease-free pectoral breast [7].

## Conclusion

Ectopic breast tissue pathologies are rarely reported, leading to a lack of awareness among clinicians regarding their presentation, diagnosis, and management. The absence of specific clinical guidelines for EBT cancer can result in misdiagnosis and inappropriate treatment, leading to poor prognosis. Clinicians should include EBT in breast examinations and consider its inclusion in screening procedures.
